# Supplementary material for: Immunization with Anaplasma centrale Msp2 HVRs Is Less Effective than the Live A. centrale Vaccine against Anaplasmosis
Source: Vaccines (Basel). 2023 Sep 29;11(10):1544. doi: 10.3390/vaccines11101544 (PMC10610995; doi:10.3390/vaccines11101544)
Supplement: Supplementary file 1 [file vaccines-11-01544-s001.zip › vaccines-2581312-supplementary.pdf]

Supplemental Table 1: Identity matrix of Msp2 HVR sequences (aa)

	Ac C	Ac G1	Ac F	Ac B1	Ac AF	Ac A1	Ac A22	V2	V3	V5	V11	V8	V1	V4	V10	V7	V9	V6	Am E6/F7	Am 1	Am 2	Am 9H1	Am G11
Ac C		53	62	42	77	51	58	50	53	56	53	52	37	39	54	53	61	60	57	41	52	41	55
Ac G1			64	45	57	52	51	39	40	44	44	41	40	41	47	48	43	57	54	40	43	41	52
Ac F				50	50	50	45	43	46	49	48	48	47	49	48	47	49	43	48	39	48	45	46
Ac B1					46	49	46	52	55	48	53	48	52	53	44	46	49	43	41	72	51	41	46
Ac AF						52	50	38	38	44	45	41	31	33	40	40	42	49	50	47	45	38	44
Ac A1							55	33	37	41	37	34	28	30	44	43	39	40	44	52	34	31	43
Ac A22								48	48	47	48	47	38	39	36	36	42	41	40	54	47	34	39
V2									92	82	87	92	78	75	50	60	80	60	46	56	89	70	65
V3										91	84	84	78	75	54	64	87	68	50	58	81	71	72
V5											76	75	69	66	59	67	90	70	58	49	73	80	75
V11												95	68	64	48	53	71	58	44	55	98	59	63
V8													70	67	49	56	72	59	45	52	97	62	64
V1														97	46	56	66	46	42	56	67	88	51
V4															49	59	69	48	44	53	63	85	54
V10																91	64	49	89	44	47	52	57
V7																	74	54	80	45	52	61	62
V9																		77	52	43	68	69	85
V6																			54	37	56	50	92
Am E6/F7																				40	43	51	46
Am 1																					55	45	42
Am 2																						59	61
Am 9H1																							55
Am G11																							
Range of identity to Ac HVR								33-50	37-55	41-56	37-53	34-52	28-52	30-53	36-54	36-53	39-61	40-60	40-57	39-72	34-52	31-45	39-55
